# Lower Inspiratory Breathing Depth Enhances Pulmonary Delivery Efficiency of ProAir Sprays

**DOI:** 10.3390/ph15060706

**Published:** 2022-06-03

**Authors:** Mohamed Talaat, Xiuhua April Si, Jinxiang Xi

**Affiliations:** 1 Department of Biomedical Engineering, University of Massachusetts, Lowell, MA 01854, USA; mohamed_talaat@student.uml.edu; 2 Department of Aerospace, Industrial, and Mechanical Engineering, California Baptist University, Riverside, CA 92504, USA; asi@calbaptist.edu

**Keywords:** inhalation therapy, metered-dose inhaler (MDI), ProAir-HFA, slow deep waveform, high-speed imaging, peak inhalation flow rate (PIFR)

## Abstract

Effective pulmonary drug delivery using a metered-dose inhaler (MDI) requires a match between the MDI sprays, the patient’s breathing, and respiratory physiology. Different inhalers generate aerosols with distinct aerosol sizes and speeds, which require specific breathing coordination to achieve optimized delivery efficiency. Inability to perform the instructed breathing maneuver is one of the frequently reported issues during MDI applications; however, their effects on MDI dosimetry are unclear. The objective of this study is to systemically evaluate the effects of breathing depths on regional deposition in the respiratory tract using a ProAir-HFA inhaler. An integrated inhaler mouth-throat-lung geometry model was developed that extends to the ninth bifurcation (G9). Large-eddy simulation (LES) was used to compute the airflow dynamics due to concurrent inhalation and orifice flows. The discrete-phase Lagrangian model was used to track droplet motions. Experimental measurements of ProAir spray droplet sizes and speeds were used as initial and boundary conditions to develop the computational model for ProAir-pulmonary drug delivery. The time-varying spray plume from a ProAir-HFA inhaler into the open air was visualized using a high-speed imaging system and was further used to validate the computational model. The inhalation dosimetry of ProAir spray droplets in the respiratory tract was compared among five breathing depths on a regional, sub-regional, and local basis. The results show remarkable differences in airflow dynamics within the MDI mouthpiece and the droplet deposition distribution in the oral cavity. The inhalation depth had a positive relationship with the deposition in the mouth and a negative relationship with the deposition in the five lobes beyond G9 (small airways). The highest delivery efficiency to small airways was highest at 15 L/min and declined with an increasing inhalation depth. The drug loss inside the MDI was maximal at 45–60 L/min. Comparisons to previous experimental and numerical studies revealed a high dosimetry sensitivity to the inhaler type and patient breathing condition. Considering the appropriate inhalation waveform, spray actuation time, and spray properties (size and velocity) is essential to accurately predict inhalation dosimetry from MDIs. The results highlight the importance of personalized inhalation therapy to match the patient’s breathing patterns for optimal delivery efficiencies. Further complimentary in vitro or in vivo experiments are needed to validate the enhanced pulmonary delivery at 15 L/min.

## 1. Introduction

The metered-dose inhaler (MDI) manual typically advises the patient to breathe slowly and deeply and synchronize the MDI actuation with the inhalation [1,2]. In reality, however, a wide variety of breathing profiles can be practiced by the patients depending on their age, gender, health, physiology, strength, or cognitive capacity [3,4]. It is widely acknowledged that many MDI users with respiratory diseases cannot completely comply with the instructions [5]. Allen et al. [6] assessed MDI usage in 30 elderly patients. Only 18 patients were able to correctly use the MDI; among them, only three inhaled ideally as instructed. Incorrect actuation timing and inadequate inhalation were the top two most frequent errors made [6,7]. 

The inhalation airflow rate is one key parameter that influences the MDI penetration efficiency. Current MDI guidelines recommend slow deep breathing at a peak inhalation flow rate (PIFR) of 60 L/min with auction during the first half of the inhalation [8]. It is noted that such guidelines were based on clinical trials using CFC (chlorofluorocarbon) MDIs and in vitro studies of HFA (hydrofluoroalkane) MDIs, which often utilized ideal inspiratory techniques and ignored the variability in breathing conditions among patients. The inability to follow the prescribed inhalation waveform is expected to noticeably change the drug delivery efficiency and associated therapeutic outcomes. 

When considering the airflow effects on MDI delivery, it should be clear that (1) there is the inhalation flow through the canister-holder space and a jet flow from the actuator orifice; and (2) the airflows can have different effects in different regions. Inside the mouthpiece, jet-induced turbulence and vortices can significantly affect drug deposition in the device and mouth. At the end of the mouthpiece, the co-flow experiences a sudden expansion from the mouthpiece to a larger oral cavity. Unique flow and aerosol behaviors in MDI spray plumes have been reported after exiting the mouthpiece. Using a laser light-sheet method, Smyth [9] observed asymmetrical patterns (ellipsoid) of the spray plumes in the vertical direction, with a time-varying elliptical ratio of 1.05–1.2. Even though the underlying reasons are still unclear, similar observations were also reported by Longest et al. [10], who termed such spray asymmetry the burst effect. They hypothesized that the burst effect could cause higher oral deposition at the start of actuation while increasing the inhalation duration could reduce the burst effect on lung deposition.

Several experimental studies have investigated the inspiratory flow-associated effects on MDI drug delivery, especially in the MDI mouthpiece and oral cavity. Fadl et al. [11] showed that a larger mouthpiece diameter moderately increased the MDI penetration efficiency into the lung. Adding a 0.5-mm-diameter wire 2 mm downstream of the actuator orifice was found to depress the turbulence inside the mouthpiece and reduce 5–10% drug loss in the device [11]. Lin et al. [12] further showed that the effect of mouthpiece diameter varied with particle size, with 2- and 8-micron particles being least affected. Biswas et al. [8] experimentally measured the lung penetration efficiency from the Ventolin-HFA inhaler under three trapezoidal inhalation waveforms. A fixed inspiratory capacity (3 L, the integrated area under each waveform) was used with varying peak inspiratory flow rates (PIFRs) of 30, 60, and 90 L/min. Higher lung deposition was reported at 60–90 L/min than 30 L/min [8]. Zhang et al. [13] measured deposition in three mouth-throat cast replicas with a Qvar inhaler at PIFR of 60 L/min and reported 25–26% in two idealized geometries and 12% in the USP duct in contrast to in vivo deposition of 29 ± 18%.

Numerical studies of the inhalation flow effects on MDI drug delivery have also been reported. Youseri et al. [14] numerically simulated the deposition of Ventolin-HFA spray droplets in an idealized mouth-throat-lung geometry extending to G1 under three constant inhalation flow rates (15, 30, 60 L/min). Results showed that most droplets were deposited in the pharynx regardless of the inhalation flow rate, and more aerosols were delivered into the lung at 30 L/min than at 15 and 60 L/min. The peak lung deposition was assumed to result from the concurrent gravitational and inertial deposition mechanisms, which were equivalent at 30 L/min, while becoming dominant, respectively, at 15 (i.e., gravitational) and 60 (inertial) L/min [14]. Kleinstreuer et al. [15] numerically simulated the drug delivery of MDIs with different propellants and predicted a lung penetration rate of 46.6% with HFA-MDI and 23.2% with CFC-MDI. Longest et al. [16] simulated the MDI deposition in a stochastic individual path model under prescribed and deviated waveforms and reported that errors in inhalation decreased the tracheobronchial dose by 30%. 

Nearly all previous studies agreed that reducing the MDI droplet size helped increase drug delivery to the lung; however, mixed results have been reported regarding the effects of the inspiratory airflow on MDI drug delivery. Fadl et al. [11] reported that reducing the inspiratory flow rate from 90 to 30 L/min tripled the delivery efficiency through a mouth-throat cast model. Yousefi et al. [14] numerically investigated the effect of the inhalation flow rate on MDI delivery and reported higher lung penetration rates at 30 L/min than at 15 and 60 L/min. On the other hand, Biswas et al. [8] recommended a peak inspiratory flow rate (PIFR) of 60–90 L/min in the first half of inhalation for asthmatic and COPD patients when using a Ventolin-HFA inhaler. 

In this study, we aimed to evaluate the dependence of the inhalation depth on the dosimetry of the ProAir spray aerosols in a combined MDI-mouth-lung geometry extending to G9. Specific aims are to (1) find the discharging velocity during ProAir actuation based on PDA-measured velocities 3 cm and 6 cm downstream of the mouthpiece, (2) record the ProAir-generated spray plume in open space using a high-speed camera, (3) model validation by a multi-time comparison between the computational and recorded results, (4) simulate ProAir deposition transport and deposition in the respiratory tract, and (5) compare the spray droplet deposition using five different breathing depths.

## 2. Results

### 2.1. Model Development for MDI Delivery

#### 2.1.1. MDI-Airway Geometry

**Figure 1:**
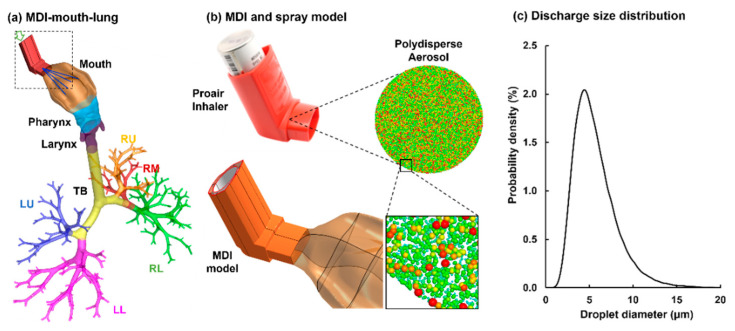
MDI delivery computational model: (**a**) an integrated MDI-mouth-lung model geometry that extends to G9, (**b**) the MDI model for ProAir-HFA and polydisperse aerosols discharged from the MDI orifice, and (**c**) the droplet size distribution typical of the ProAir-HFA inhaler.

To study the patient-device interactions, an integrated MDI-airway geometry was constructed (Figure 1a). The MDI inhaler was modeled after an actual ProAir-HFA inhaler (upper panel, Figure 1b). The airway model extended from the mouth-opening to lung bronchioles of the ninth generation of bifurcations (i.e., G9). This model was developed in our previous studies, with detailed procedures reported in [17,18,19]. The shape of the mouth opening was adapted to connect snuggly with the inhaler mouthpiece. The mouth-lung geometry was separated into multiple regions to quantify regional dosages, which included the mouth, pharynx, larynx, tracheobronchial region (TB), and five lobes: right upper (RU), right middle (RM), right lower (RL), left upper (LU), and left lower (LL), as displayed in Figure 1a.

#### 2.1.2. Modeling of Spray Aerosols during Actuation 

To model the MDI actuation, a bolus aerosol was released from the MDI orifice, which had a diameter of 0.4 mm. The size distribution of the discharged aerosol droplets followed the measurement by Liu et al. [20], which had a volume median diameter Dv50 of 5.2 µm and a variation of 0.4 µm, as illustrated in Figure 1b,c.

#### 2.1.3. Inhalation Waveforms: Control and Variants

**Figure 2:**
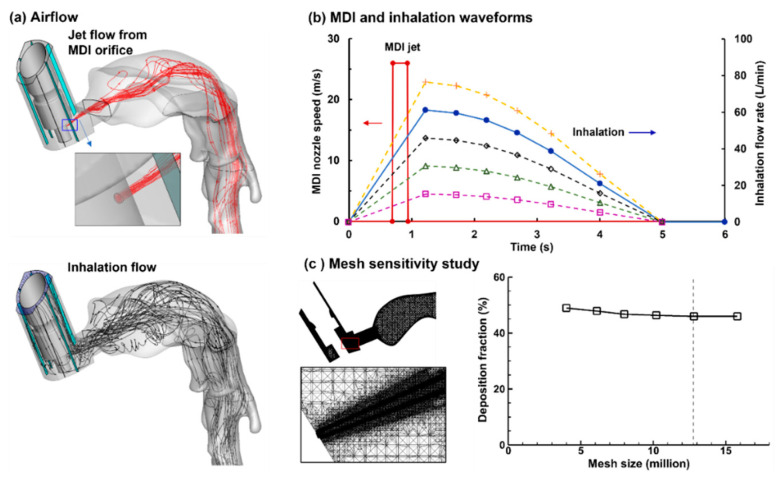
Airflow and computational mesh: (**a**) there are two airflows during MDI delivery: an excipient flow from the MDI orifice (upper) and an inhalation flow by the patient, (**b**) five inhalation waveforms with the peak inhalation rate ranging from 15 to 75 L/min, and (**c**) computational mesh and grid-independent study.

There were two airflows during the MDI usage, the jet flow from the MDI orifice (Figure 2a, upper panel) and the inhalation flow by the patient (Figure 1a, lower panel). The MDI jet flow at the orifice had a discharge velocity of 26 m/s, which started at 0.72 s after inhalation and lasted for 0.23 s. The flow through the MDI holder-canister space followed a slow-deep (SD) waveform with a peak flow rate of 60 L/min and a duration of 5 s to mimic the patient’s inhalation [16]. To evaluate influences from the inspiratory breathing depth on MDI dosimetry, four more inhalation waveforms were evaluated, which were uniformly scaled up or down to have peak inspiratory flow rates of 75, 45, 30, and 15 L/min, respectively (Figure 2b). 

#### 2.1.4. Computational Mesh

To sufficiently resolve the multiscale geometries in this study, multi-domain computational meshes were generated that consisted of ultrafine mesh downstream of the nozzle orifice, coarse mesh in the upper airway, and fine mesh in the bronchioles. To ensure grid-independent predictions, a sensitivity study of computational mesh was conducted by comparing the regional deposition fraction (DF) among six mesh sizes ranging from coarse (3.2 million) to very fine (16 million), as illustrated in Figure 2c. Considering that the near-wall cell mesh quality is critical in numerical accuracy in predicting micrometer aerosol deposition [21], all of the six meshes consisted of five layers of body-fitted prismatic cells, with the first layer cell height being 0.05 mm. Grid-independent results were safely achieved with a mesh size of 12.8 million (Figure 2c), which was used for all subsequent simulations. 

### 2.2. Characterization of MDI Actuation

#### 2.2.1. Imaging of MDI Sprays

**Figure 3:**
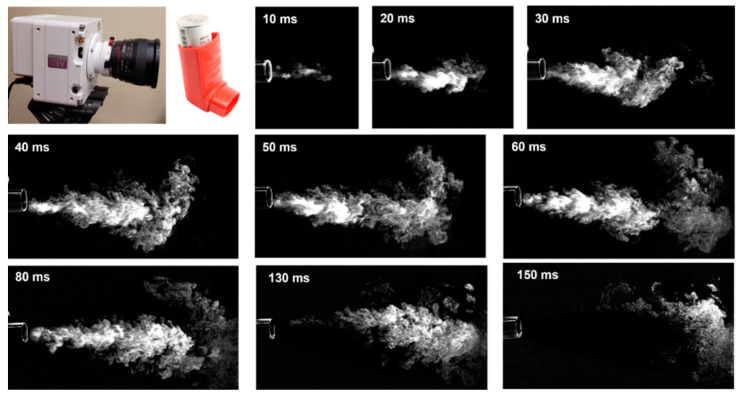
High-speed imaging of the MDI spray plume at varying instants after being discharged into an open space (10–150 ms).

The temporal evolution of the MDI spray plume was shown in Figure 3 during 10–150 ms after actuation. A salient feature was the irregular outskirts of the spray plume, indicating an intensified vortex formation and significant mixing between aerosol droplets and ambient air. This led to the slow speeds of the spray droplets and airflow vortices at the outskirt and front of the jet, in contrast to the high speed of the jet core. A slightly downward angle of the spray plume was noted (30–90 ms), which might result from a downward mouthpiece orientation or gravitational sedimentation.

**Figure 4:**
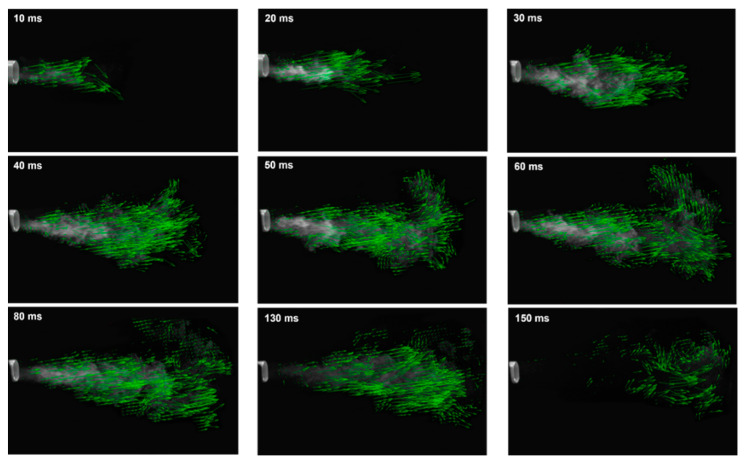
PIVlab-analyzed velocity fields of the MDI spray plume at varying instants after actuation (10–150 ms).

The vector fields of the MDI spray plume at varying instants after actuation were obtained by applying PIVLab analysis to the high-speed recording and are shown in Figure 4. It is clear that the advancement of the spray plume was not linear vs. time and that vortex formation was strong at the jet flow outer boundary. The vortices were particularly clear at T = 150 ms when the jet flow decayed almost completely (Figure 4, last panel). The speed of the spray jet fluctuated in the range of 6–15 m/s during 10–30 ms. At a distance of 3 cm from the mouthpiece, the jet speed was found to be around 9.7 ± 2 m/s.

#### 2.2.2. Reverse Identification of Orifice Discharge Velocity 

**Figure 5:**
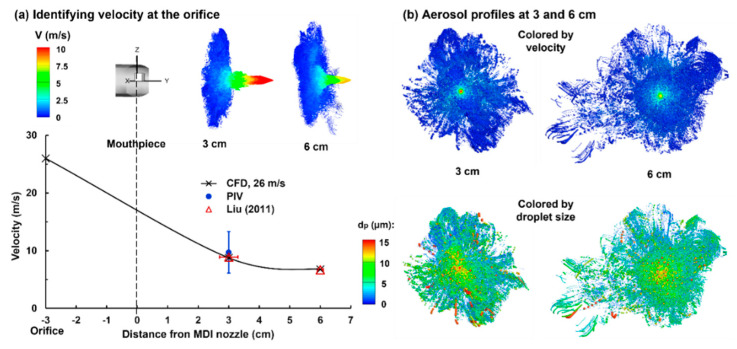
Reverse identification of the discharge velocity at the MDI orifice in comparison to experimental measurements: (**a**) identifying the velocity at the orifice using LES predictions and (**b**) predicted aerosol profiles at 3 and 6 cm from the mouthpiece.

To identify the orifice discharge velocity, a series of trails of guessed velocity magnitudes were conducted in order to match the PDA measurements at 3 cm and 6 cm away from the mouthpiece by Liu et al. [20]. Figure 5a shows the LES-Lagrangian predicted results with an orifice speed of 26 m/s. Good agreement was obtained between the numerical and experimental spray speeds at both 3 cm and 6 cm from the mouthpiece. In particular, at 3 cm, the PIVLab-analyzed speed based on the high-speed recording also agreed well with both the predicted and measured results. By plotting the scatter-vector profiles of the spray aerosols at 3 cm and 6 cm (Figure 5, upper panel), the fast-moving jet core exhibited an apparent contrast to the slow-moving plume outskirts. Furthermore, the jet core velocity decreased by approximately 23%, which was consistent with the measurements by Liu et al. [20].

The aerosol profiles were further plotted in Figure 5b and were colored either by the speed (upper panel) or droplet size, where the scatter sizes were scaled to the individual droplet size (lower panel). A quick decrease in the droplet speeds was observed from the center to the periphery of the spray plume (Figure 5b, upper panel). In addition, more large droplets were noted in the lower spray plume due to gravitational sedimentation (Figure 5b, lower panel).

**Figure 6:**
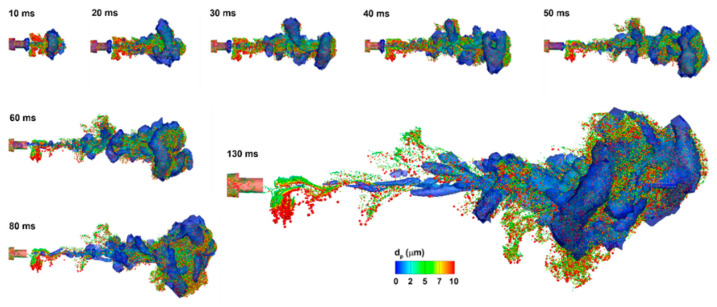
MDI vortex and aerosol dynamics at varying instants after being discharged.

Figure 6 shows the temporal variation of the predicted spray vortex and droplet dynamics after being released into an open space. Again, there was enhanced mixing between the aerosol and ambient air at the outskirts and front of the spray plume. We also noticed the resemblance between the instantaneous vortex structures and the aerosol topologies, indicating a strong association between the vortex and discrete-phase dynamics. This also highlighted the need to accurately capture the temporal evolution of these vortices, whenever feasible, to accurately capture the aerosol motions and the subsequent dosimetry distributions. Subtle differences between the vortices and aerosols were also observed, for instance, at 130 ms, with droplets lagging behind the vortices. These, however, were also expected, considering the quick response of air versus the slow responses of liquid droplets (i.e., longer aerosol residence time).

### 2.3. Flow and Aerosol Dynamics in Airway: Control Case (Q_Max_ = 60 L/min)

#### 2.3.1. Airflow Dynamics

**Figure 7:**
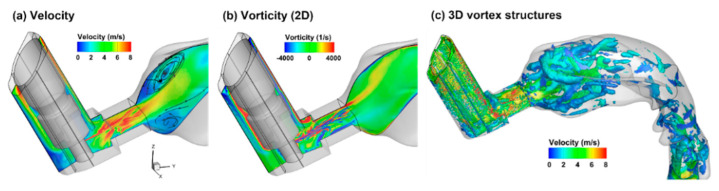
Airflow features inside the MDI and mouth 100 ms after MDI actuation under the inhalation waveform of Qmax = 60 L/min: (**a**) velocity, (**b**) vorticity, and (**c**) 3D vortex structures.

Very different airflow dynamics were predicted when applying the MDI spray into a mouth cavity and into an open space (Figure 7 vs. Figure 6). The inhalation waveform of the patient had a peak inhalation flow rate of 60 L/min (i.e., control case in Figure 2b). Even though the jet flow still prevailed inside the mouthpiece in both cases, the flows in the mouth cavity varied notably. Two recirculating zones formed close to the mouth roof and floor in the front mouth because of the abrupt expansion from the inhaler mouthpiece. The intensified shear flows in the font mouth also led to stronger vortex formation than in other regions except for the larynx (Figure 7c). As a result, elevated dispersion of aerosol droplets was also expected in the front mouth cavity and the larynx.

#### 2.3.2. Spray Droplet Dynamics

**Figure 8:**
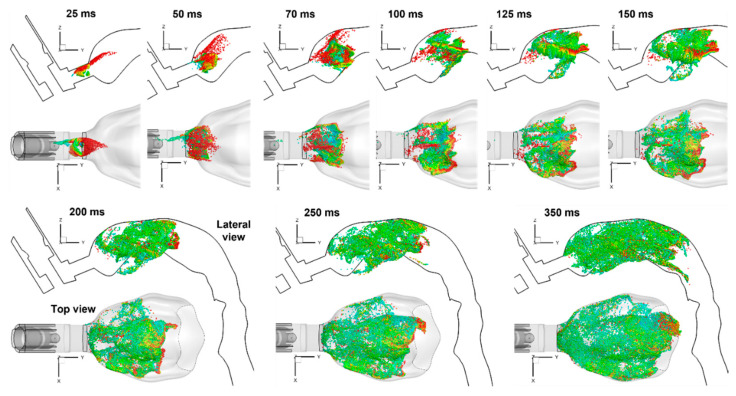
Snapshots of spray aerosols with lateral and top views at varying instants after MDI actuation at Q_max_ = 60 L/min. The aerosol symbols are colored according to their individual diameters.

Likewise, very different aerosol behaviors in the mouth were predicted than those discharged into an open case (Figure 8 vs. Figure 6). Instead of a jet that persisted for a long distance (Figure 6), the ProAir-HFA spray droplets became dispersed nearly immediately, leaving the mouthpiece. A recirculating motion of the droplets was observed during 125–250 ms. The droplets spread over the entire mouth cavity at around 350 ms. After that, the inspiratory flow gradually dispensed these droplets downstream to the lower airway (i.e., the dispense phase).

### 2.4. Effects of Breathing Depth 

#### 2.4.1. Variation in Airflows

**Figure 9:**
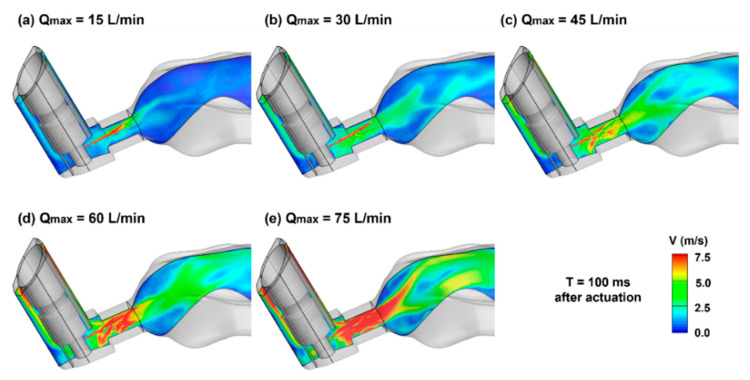
Mid-plane velocity contours at 100 ms after actuation with varying breathing depths (Q_max_): (**a**) 15 L/min, (**b**) 30 L/min, (**c**) 45 L/min, (**d**) 60 L/min, and (**e**) 75 L/min.

To investigate the influences of the patient’s breathing depths during MDI application, the velocity contours on the middle plane are compared in Figure 9 at 100 ms after actuation. It is noted that during the MDI actuation (T = 0.72 s), the volumetric flow rate ranged from 9 to 45 L/min for the five test cases, while that for the orifice jet was 0.47 L/min. The variation of the inhalation significantly altered the main flows, as well as their interactions with the orifice jet in both the MDI mouthpiece and mouth cavity. Accordingly, significantly different behaviors and fates of entrained spray droplets in the respiratory tract were expected for different breathing conditions.

#### 2.4.2. Surface Deposition of MDI Droplets

**Figure 10:**
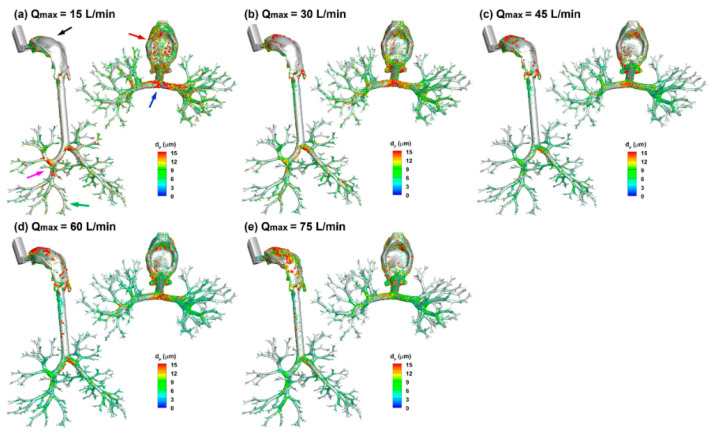
Surface deposition of MDI sprays for varying breathing depths (Q_max_): (**a**) 15 L/min, (**b**) 30 L/min, (**c**) 45 L/min, (**d**) 60 L/min, and (**e**) 75 L/min.

Figure 10 shows the deposition distribution of ProAir spray droplets with different breathing depths. Several differences are worth noting and are reflective of the inhalation-dependent transport and deposition mechanisms of the spray droplets. First, at a lower breathing depth (Q_max_ = 15 L/min), low deposition occurred on the mouth roof (black arrow) and high deposition on the mouth floor (red arrow). This was because of the relatively higher gravitational sedimentation than the inertial impaction in the mouth-throat region and the convection in the downstream airway. 

As the inhalation increased (i.e., Q_max_ from 15 to 75 L/min), the deposition on the mouth roof progressively increased, while that on the mouth floor progressively decreased. Note that the trachea and lung in this study are inclined forward by approximately 15°, which represented a position that the patient typically took during MDI drug delivery. The second notable difference occurred at the main and second carina ridges (blue and pink arrows, Figure 10a), which had high doses at Q_max_ = 15 L/min and decreased with increasing breathing depth (Figure 10a–e). Different deposition patterns were also observed in the lower lobes (G4–G9, green arrow in Figure 10a). However, the trend with the breathing depth was not apparent.

#### 2.4.3. Deposition Fraction (DF) and Penetration Rate (PR) vs. Time

**Figure 11:**
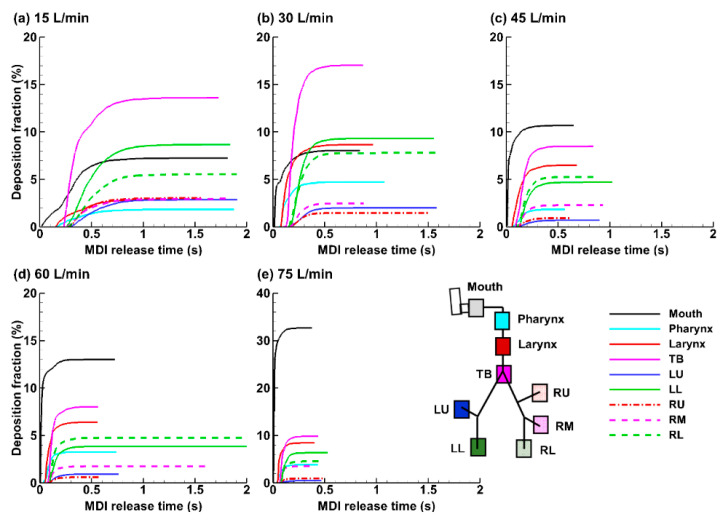
Temporal evolution of the regional deposition fraction (DF) under varying breathing depths (Q_max_): (**a**) 15 L/min, (**b**) 30 L/min, (**c**) 45 L/min, (**d**) 60 L/min, and (**e**) 75 L/min.

The temporal evolution of the droplet deposition in different regions of the mouth-lung geometry after the MDI actuation is shown in Figure 11a–e for the five breathing depths considered. Deposition in the mouth started immediately after the MDI actuation. Its magnitude was highly sensitive to the breathing depth, which was 7.2% at Q_max_ = 15 L/min and increased to 32.7% at Q_max_ = 60 L/min (see black lines, Figure 11a–e). An abrupt increase occurred when the flow rate increased from 60 to 75 L/min, i.e., from 13.0% to 32.7%, which was more than doubled, indicating either an escalating inertial impaction, turbulent effect, or both, at 75 L/min. Note that the spray has a polydisperse size distribution and the mass of a droplet is proportional to (d_p_)^3^. For 45–75 L/min, the drug loss in the mouth was the highest among all regions (Figure 11c–e). The drug loss in the mouth was greatly reduced at lower breathing depths of 15 and 30 L/min; by contrast, more doses were observed in the TB region (pink line, Figure 11a,b).

**Figure 12:**
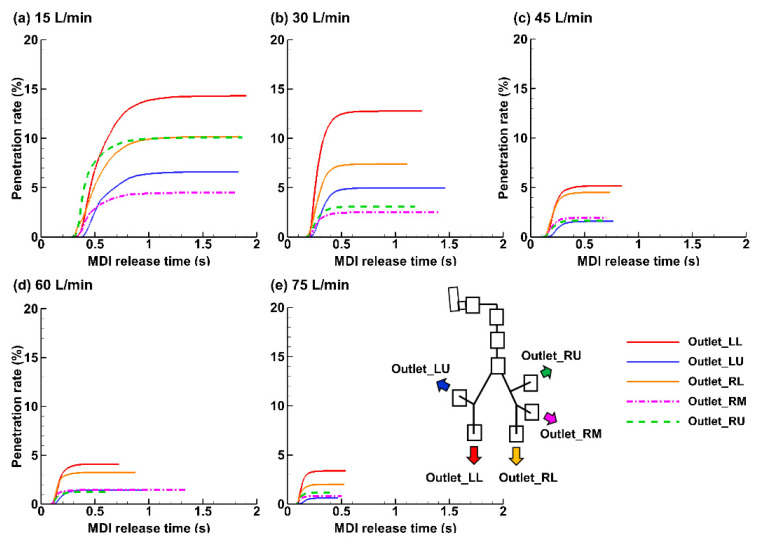
Temporal evolution of the penetration rate into peripheral lungs under varying breathing depths (Q_max_): (**a**) 15 L/min, (**b**) 30 L/min, (**c**) 45 L/min, (**d**) 60 L/min, and (**e**) 75 L/min.

Figure 12 shows the penetration rate of the applied MDI spray into the five lung lobes beyond G9. Visualized under the same scale (i.e., 0–20%), it is clearly shown that the dose into the lower airway decreases with increasing inhalation flow rate. The total penetration rate (PR) beyond G9 was 45.7% for 15 L/min, 30.7% for 30 L/min, while only 14.9%, 11.5%, and 8.0% for 45, 60, and 75 L/min, respectively. At 15 L/min, the individual lobar PR in the left lower (LL) was 3.5 times that at 60 L/min (i.e., 14.3% over 4.1%) and 4.2 times that at 75 L/min (i.e., 14.3% over 3.4%). This finding, however, should not be stretched to conclude that the lower the breathing depth, the higher the doses into the pulmonary regions; these aerosols still need sufficient time to reach the deep lung and deposit there by sedimentation. As such, a slow and deep waveform with a low peak-inhalation-flow rate of 15 L/min should deliver the optimal dose of ProAir sprays into the lungs. The two lower lobes (right lower: RL and LL) received higher doses than the two upper lobes (right upper: RU and left upper LU), regardless of the breathing depth. This was presumably attributed to a concurrent influence from inertia and gravity, both of which pointed to the lower lobes. The PR to the RL and LL lobes was 2–3 times that of the RU and LU lobes.

**Figure 13:**
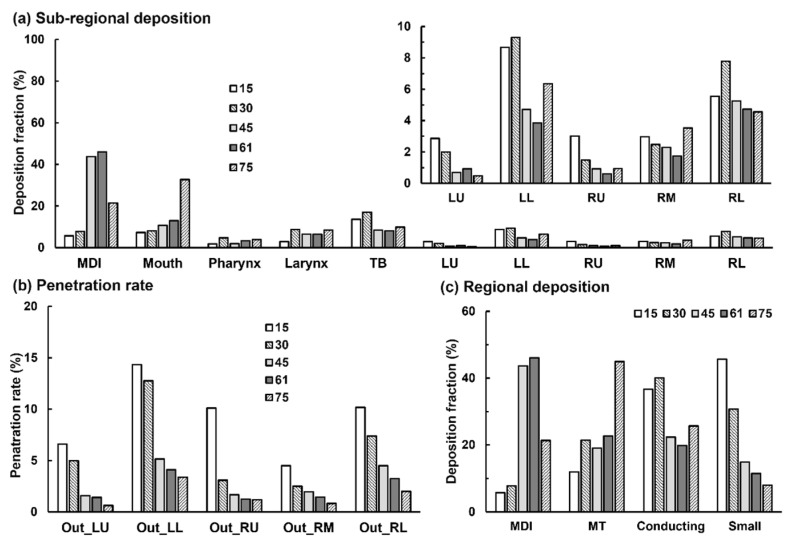
Dosimetry comparison among varying breath depths: (**a**) sub-regional DF till G9, (**b**) penetration rate beyond G9, and (**c**) DF in the device, mouth-throat, conducting lung, and small airways.

The final dosimetry distributions in different airway regions were compared in Figure 13 among the five breathing depths, with Figure 13a summarizing the sub-regional DF and Figure 13b the penetration rate into five lobes beyond G9 (small airway). The drug loss inside the MDI itself was also included in Figure 13a. Interestingly, the highest MDI losses were predicted at Q_max_ = 45 and 60 L/min, which decreased at 75 L/min. This may be because more inhalation air envelopes the orifice jet and blocks the aerosols from reaching the mouthpiece wall. 

The sub-regional deposition and penetration rates were further grouped into four regions: device (MDI), mouth-throat (MT, or upper airway including mouth, pharynx, and larynx), conducting airway (TB and five lobes up to G9, or large airway), and small airway (five lobes beyond G9). It is observed that the PIFR of 15 L/min resulted in the lowest drug loss in the device and oropharynx, high deposition in the conducting airway, and the highest deposition in the small airway among the five breathing depths considered. The delivery efficiency to the small airway was predicted to be 45.7% at PIFR of 15 L/min, which was 1.5 times that at 30 L/min (30.7%), 3.1 times that at 45 L/min (14.9%), 4.0 times at 60 L/min (11.4%), and 5.7 times that at 75 L/min (8.0%). 

This observation underscored the high sensitivity of pulmonary drug delivery to the inhalation flow rate. Considering that young and elderly patients usually cannot adequately adhere to the best practice instructions, the variation can cause drastically different delivery efficiencies to the deep lung. It is also noted that the cumulative deposition in the conducting lung (TB-G5) is much less sensitive to the breathing depth, particularly for 45–70 L/min (Figure 13c). 

#### 2.4.4. Deposition Enhancement Factor (DEF)

**Figure 14:**
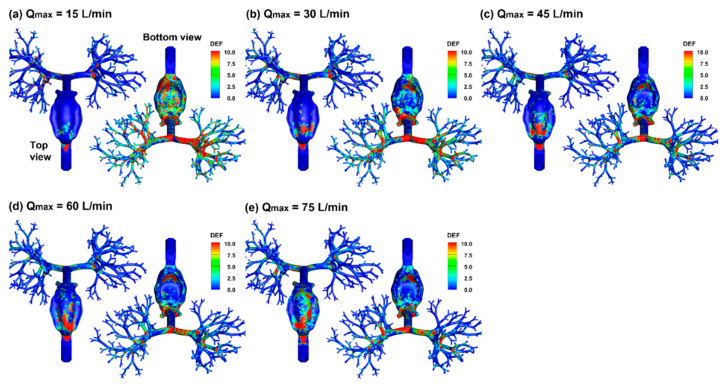
Comparison of the local dosimetry (DEF: deposition enhancement factor) among varying breath depths (Q_max_): (**a**) 15L/min, (**b**) 30 L/min, (**c**) 45 L/min, (**d**) 60 L/min, and (**e**) 75 L/min.

Figure 14 visualizes the local dosimetry in terms of DEF (deposition enhancement factor), which is defined as the ratio of the local DF over the averaged DF. Compared to the surface deposition in Figure 10, DEF quantifies the dose accumulation by summing all deposited droplet masses of varying diameters in local regions and thus will not be affected by the droplet overlapping or droplet size. Despite highly heterogenous DEF distributions, there is a clear trend that with increasing breathing depth, the local dose on the mouth roof increases (top view), while that on the ventral wall of the respiratory tract gradually decreases (bottom view). This trend is reflective of a more dominating gravitational deposition mechanism at 15 L/min, which gradually switches to convection and inertia impaction as the inhalation increase from 15 to 75 L/min. There are more doses in the lung under lower inhalation depths, as demonstrated by the higher intensity and larger extent of DEF at 15 L/min than those at 75 L/min (Figure 14a vs. Figure 14e).

## 3. Discussion

In this study, we observed a peak drug loss in the MDI around 45–60 L/min, which increased with the inhalation flow rate from 15 to 45 L/min and decreased from 60 to 75 L/min (Figure 13c). This modal profile might result from two counteracting deposition mechanisms associated with breathing depth: (1) a quicker inhalation will intensify turbulence in the mouthpiece, yielding a higher device loss; on the other hand, (2) the inhalation flow envelops the orifice jet flow; when the flow rate is larger than 60 L/min, the envelope effect outweighs the jet-induced turbulent effect, which decreases the drug loss by blocking the spray aerosols from reaching the mouthpiece wall. In the oral cavity, the deposition is controlled by inertial and gravitational sedimentation. A quicker inhalation increases the droplet inertia and thus increases the drug loss in the mouth-throat (MT) region; thus, the MT deposition increases drastically from 60 to 70 L/min due to the quick increase in inertia. By contrast, gravitational deposition is the more dominating mechanism at 15 L/min, which gradually switches to convection and inertia impaction as the inhalation increases from 15 to 75 L/min. In the conducting lung (TB-G9), the deposition was higher at 15–30 L/min due to the more predominant gravitational mechanism at lower flow velocities. 

The delivery efficiency was predicted to increase with decreasing inhalation flow rate to both the conducting lung and small airway (Figure 13c). This was consistent with the experiments by Fadl et al. [11], who reported a tripled MDI PR to the lung when the inhalation flow rate decreased from 90 to 30 L/min. Similarly, we predicted a 3.8-times increase in lung penetration rate (beyond G9) from 75 to 30 L/min and a 5.7-times increase from 75 to 15 L/min. It is noted that the inhaler used in Fadl et al. [11] was a Proventil-HFA (3M Pharmaceuticals, St. Paul, MN, USA), whose spray size distribution and speed were similar to those of the ProAir-HFA used in this study [20]. Moreover, the mouth model hereof was reconstructed from the cast used in Fadl et al. [11], both of which were originally from Cheng et al. [22]. The definition of ‘lung’ in Fadl [11] includes both the conducting and small airway. 

In contrast to the consensus that reducing MDI droplet size helped increase drug delivery to the lung, mixed results have been reported in previous studies regarding the effects of the inspiratory flow rate. Yousefi et al. [14] numerically investigated the effect of the inhalation flow rate on MDI delivery and reported higher lung penetration rates at 30 L/min than 15 and 60 L/min. Two reasons might cause the differences between Yousefi et al. and this study. First, the MDI simulated in [14] was Ventolin-FHA, which was specified to have a mean diameter of 16.54 µm and an initial speed of 100–120 m/s. These were significantly different to those from a ProAir-HFA (mean diameter: 4.2 µm, initial speed: 26 m/s) [20]. Second, steady inhalation flow was simulated in [14], while a transient inhalation was simulated in this study, with a 5-s waveform and a 0.2-s actuation of jet flow-aerosol from 0.72 s after inhalation. The temporal and spatial variations in airflow and aerosol dynamics (Figure 8 and Figure 9), as well as the resultant discrepancies in dosimetry (Figure 10, Figure 11, Figure 12 and Figure 13), highlighted the importance of including interactions among the inhalation flow, orifice jet flow, orifice spray aerosols, and respiratory tract. Moreover, the upper airway geometries are different: an idealized model adopted from Cheng et al. [22] was used by Yousefi et al., which was composed of circular cross-sections with varying diameters [14]. In this study, a more realistic airway geometry was used that was also reconstructed from Cheng et al. [22], but retained all anatomical details, such as the tongue, the lip-tongue space, and the lumen between the teeth and cheeks [23]. Longest and Xi [23] compared the dosimetry differences between oral models of varying complexities (i.e., realistic, elliptic, circular, and constant-diameter) and revealed significant differences in deposition distributions among models, with the realistic model providing the closest approximation to complemental experiments. This may also explain why the highest deposition in the mouth-pharynx-throat was predicted in the mouth in this study vs. in the pharynx in Yousefi et al. [14]. 

In another study, Biswas et al. [8] experimentally studied dosimetry from a Ventolin-HFA MDI in the Alberta Idealized Throat model under real-life inspiration waveforms typical of asthmatic and COPD patients. Contrary to our and Yousefi et al.’s results, Biswas et al. [8] reported a moderately but consistently higher lung penetration rate at 60–90 L/min than 30 L/min. There exist at least two differences in the test conditions that may contribute to the apparent different conclusions between Biswas et al. and ours: (1) different devices (Ventolin-HFA vs. ProAir-FHA), and (2) different inspiratory capacities (IC) (a fixed IC regardless of the flow rate in Biswas et al. [8] vs. a variable IC scaled with PIFR in ours). The factors leading to different results between Biswas et al. [8] and Yousefi et al. [14] are more subtle because the same type of inhaler (Ventolin-HFA) was considered in both studies. These included: (1) different inhalation conditions (transient vs. constant), and (2) the accuracy of modeling spray properties. The mean diameter of 16.54 µm and an initial speed of 100–120 m/s were used for discharged Ventolin sprays [14], which are significantly higher than the mean diameter of 9.1 µm measured by Liu et al. [20], and the initial velocity of 40 m/s predicted by Talaat et al. [19]. The mouth-throat models in Biswas et al. [8] and Yousefi et al. [14] are also different, even though they may not be significant contributors to their observed differences. The Alberta Idealized Throat model used by Biswas et al. was a population-based idealized geometry and was manufactured from steel [24], while the computational model in Yousefi, as well in this and Fadl et al.’s studies, was originally from an oral silicon compression in Lovelace Respiratory Research Institute (i.e., LRRI cast) [22]. By the way, considering different material properties, Zhou et al. [25] experimentally compared aerosol deposition in the Alberta and LRRI casts and observed a larger amount of rebounded aerosols in the Alberta metal cast without coating treatment. 

Overall, a comparison between different MDI delivery studies required the specification at least of the atomized spray properties (aerosol size and speed), inhalation condition, and airway geometry. We wish to acknowledge that each of the above studies is a valuable stepstone that leads us closer to the real situation during the spray actuation and delivery. The mixed results and apparently different conclusions also emphasize the high level of sensitivity of MDI dosimetry to operating parameters, as well as the challenges of accurately predicting the pulmonary drug delivery from MDIs.

The high dependence of lung deposition on inhalation depth implies that matching the right patient with the right inhaler is key to ensuring effective drug delivery and good compliance [26]. Elderly patients on inhalation therapy should be properly instructed by the prescribing doctor [6]. The overall increase of lung deposition with lower inhalation depth hints that ProAir can be an appropriate option for asthma and COPD patients, whose inspiratory capacity can be impaired and inadequate inhalation can be a common problem [27]. The unintentionally lower inhalation depth executed by an asthmatic or COPD patient may deliver equivalent or even better doses to the lung compared to the current recommended breathing maneuver. This inadvertent advantage, however, should be further verified using either in vitro, in vivo, or both studies. 

The selection of drug delivery device and protocol depends on the target site. The asthmatic lesion sites can be in the conducting lungs or the small airways, while COPD occurs predominantly in small airways. In this study, we observed that the ProAir delivery efficiency to small airways is very sensitive to the PIFR and that a PIFR of 15 L/min was 45.7%, which was 1.5 times that at 30 L/min (30.7%) and 4.0 times that at 60 L/min (11.4%). As a result, a slow breathing profile with the PFRA at 15 L/min would be adopted by COPD patients. Considering that COPD patients often experience breathing difficulties and have more difficulties in executing forced respiration, a lower PIFR will make it easier for the patient to follow the instructed waveform and achieve the desired delivery efficiency. This also implies that the seemingly inadequate inhalation that had often been practiced unintentionally by some COPD or asthmatic patients may even deliver a higher order to the small airways than the current ideal technique when using a ProAir inhaler. 

The major limitations of this study are the neglect of (1) evaporations and collisions among the spray droplets, (2) electrostatic charges remaining from the atomization process, and (3) airway compliance. Droplets can change their sizes from evaporations and collisions, which can alter the drug deposition distribution [28,29,30]. These effects can be more significant with high volatility propellants and solvents [31]. The electrostatic charges on the droplet surface can also modify the droplet trajectories by attraction, repulsion, image force from the negatively charged mucosa, and electric space force if an external electric field exist [32,33,34,35]. The respiratory tract expands noticeably during deep breathing, particularly the glottis and the lung [36,37,38]. However, these factors are assumed to be secondary in comparison to the major factors, such as the spray size distribution and inhalation flow rate, as investigated in this study. Moreover, the results of this study were only partially validated by comparing the spray developments in the open space with high-speed images, while the deposition in the airway was not validated due to the lack of comparable data in the literature. The mouth-lung geometry herein was representative of a healthy subject, whereas respiratory diseases, such as asthma, can modify the flow field and lead to different drug deposition distributions. Therefore, future complementary experimental tests in human subjects or airway replicas with relevant respiratory diseases are needed for either model validation or dosimetry estimations in patients. 

## 4. Materials and Methods

### 4.1. MDI and Airway Models 

The MDI model was built in SolidWorks based on a ProAir-HFA inhaler. The mouth cavity was reconstructed from an oral impression cast [39]. The pharynx and larynx were reconstructed based on computerized tomography (CT) images of a 53-year-old female [23]. The lung geometry was generated mathematically using an in-house module *Lung4Cer* [40,41,42,43]. It extended from the trachea to the ninth generation (G9) of lung bifurcation and had 512 bronchiolar outlets. A forward slope of 15° was retained for the trachea to represent its natural orientation when a subject took an upright position. 

### 4.2. High-Speed Imaging and Image Analysis

To understand the discharge and dispersion of the spray plume from the ProAir-HFA inhaler, a high-speed camera (Phantom VEO 1310) with an acquisition speed up to 11,000 fps was used to record the actuation process. A laser sheet of 488 nm (OXlaser, 100 mW) was used to illuminate the spray plume. Different acquisition speeds (2000–6000) were tested and compared to obtain the optimal imaging of the release and evolution of the MDI sprays. The high-speed images were subsequently analyzed using PIVLab software to quantify the spray plume speeds [44].

### 4.3. Numerical Methods 

#### 4.3.1. Boundary Conditions 

Isothermal and incompressible flows were assumed for the inhaled air. The waveform for patient inhalation and MDI actuation followed two separate waveforms. Typically, a slow deep breathing maneuver (control case) would be practiced during MDI drug delivery, which had a peak inhalation flow rate Q_max_ of 60 L/min and lasted for 5 s [16]. In order to evaluate the breathing depths on MDI delivery efficiency, four more waveforms were considered in this study by uniformly scaling up and down the control-case waveform, i.e., with the Q_max_ being 75, 45, 30, and 15 L/min. The first two waveforms represented the variance of the control base (i.e., Q_max_ = 60 ± 15 L/min), while the other two (i.e., Q_max_ = 30 and 15 L/min) represented the scenario where the patient could not perform the prescribed breathing maneuver due to impaired breathing capacity due to respiratory diseases. The flow from the MDI orifice followed a step-shaped waveform, which was actuated 0.72 s from the start of the inhalation and lasted 0.2 s. The velocity of the jet flow and spray droplets within the flow was determined numerically using a trial-and-error manner to match the PDA measurements [20], with more details given in the following section. 

#### 4.3.2. Experiment-Based Estimation of the Spray Discharge Speed 

To numerically study the patient-device interactions, the discharge velocity at the MDI orifice is needed. However, reports that measured the ProAir discharge speed at the orifice were not found yet. Rather, spray speeds downstream of the orifice were measured, for instance, at 3 cm and 6 cm from the mouthpiece by Liu et al. using phase Doppler anemometry (PDA) [20]. A series of numerical experiments with guessed discharge velocities were conducted by Liu. that aimed to match the existing measurements at 3 cm and 6 cm from the mouthpiece [20]. The discharge velocity at the orifice would be identified when the predicted and measured spray speeds match at both locations. 

#### 4.3.3. Flow and Particle Transport Simulations 

Large-eddy simulations (LES) with wall-adapting local eddy-viscosity (WALE) model were used to simulate the airflow flows. This method has been shown to be able to accurately capture the laminar-turbulent transitions [45]. The transport and deposition of MDI spray droplets were tracked using a discrete-phase Lagrangian method. To simulate the spray releasing from the ProAir-HFA inhaler, 100,000 droplets were discharged from the 0.4-mm-diameter nozzle orifice with a velocity of 26 m/s. The size distribution of the discharged aerosol droplets followed the measurement by Liu et al. [20], which had a volume median diameter Dv50 of 5.2 µm and a variation of 0.4 µm, as illustrated in Figure 1b,c. The airway walls were rigid and had a no-slip condition (i.e., *u_wall_* = 0). Wall deposition was assumed whenever droplets touched the wall. A user-defined function was implemented in the near-wall region to account for the velocity transition from the near-wall cell to the wall, which had been shown to yield improved predictions for both nano- and micro-meter particle deposition [46].

Ansys Fluent 21 (Canonsburg, PA, USA) was used to solve the airflow and droplet transport governing equations. Ansys ICEM CFD was used to generate the computational mesh. Considering the multi-scale feature of the model geometry (i.e., 0.4-mm diameter at the nozzle orifice, 40-mm width in the oral cavity, and 1.5-mm diameter at bronchiolar outlets), a multi-domain mesh was generated with ultrafine mesh downstream of the orifice, coarse mesh in the upper airway, and fine mesh in the lower lung (Figure 2c). Prism elements were generated in the near-wall region to capture the boundary layer flows. Body-fitted prism elements were generated in the near-wall region [46]. Because a polydisperse distribution was adopted for spray aerosols, the deposition fraction (DF) was calculated via post-processing based on the deposited and applied mass:

(1) $$DF_{i}=\frac{\sum_{j=1}^{N}\rho_{p}\left(\pi d_{j}^{3}/6\right)}{Total\;mass\;of\;seed\;particles}$$

Here *i* is the sub-region index, *j* is the droplet index, *N* is the number of droplets deposited in the region *i*, and *ρ_p_* and *d_j_* are the droplet density and diameter. To characterize the local dosimetry, the deposition enhancement factor (DEF) was calculated as the ratio of the DF on a local area (i.e., a 0.5-mm-diameter circle) over the mean DF:

(2) $$DEF_{i}=\frac{DF_{i}/A_{i}}{Total\;mass\;of\;seed\;particles/\;total\;area\;of\;the\;airway}$$

## 5. Conclusions

A computational model was developed for pulmonary drug delivery with ProAir-HFA using experimental measurements as initial/boundary conditions for the spray aerosols at the actuator orifice. The effects of inhalation depth on ProAir inhalation dosimetry were evaluated in a mouth-G9 geometry by scaling the peak flow inhalation flow rate (PIFR) of a slow-deep waveform from 60 to 15, 30, 45, and 75 L/min. Specific findings include:

1. The initial velocity of the spray plume from the ProAir-FHA actuator orifice was predicted to be 26 m/s, which matched the measured velocities at 3 and 6 cm from the mouthpiece.
2. The LES-Lagrangian predicted spray plume topologies into the open-air agreed well with corresponding high-speed images both temporally and spatially.
3. The drug loss in the device itself peaked at 45–60 L/min, while that in the mouth constantly increased with the inhalation depth from 15 to 75 L/min.
4. The pulmonary drug delivery efficiency (beyond G9) had a negative relationship with the inhalation rate, with a predicted penetration rate of 11.4% at PIFR of 60 L/min, 30.7% at 30 L/min, and 45.7% at 15 L/min.
5. Model cross-validation with existing experiments indicates a high dosimetry sensitivity to initial spray properties (size and velocity) and transient inhalation rate, which should be correctly considered in numerical modeling for accurate dosimetry predictions.
6. This study has not reached the most efficient way of inhalation for optimal ProAir delivery yet; however, improved personalized inhalation therapy can be achieved by matching the inhaler type with the patient’s disease and breathing capacity.

## Data Availability

Data are available within the article.
